# Lattice Strain Mapping of Platinum Nanoparticles on Carbon and SnO_2_ Supports

**DOI:** 10.1038/srep13126

**Published:** 2015-08-18

**Authors:** Takeshi Daio, Aleksandar Staykov, Limin Guo, Jianfeng Liu, Masaki Tanaka, Stephen Matthew Lyth, Kazunari Sasaki

**Affiliations:** 1 International Research Center for Hydrogen Energy, Kyushu University, Fukuoka 819-0395, Japan; 2 Next-Generation Fuel Cell Research Center (NEXT-FC), Kyushu University, Fukuoka 819-0395, Japan; 3 Faculty of Engineering, Department of Hydrogen Energy Systems, Kyushu University, Fukuoka 819-0395, Japan; 4 International Institute for Carbon-Neutral Energy Research (WPI-I2CNER), Kyushu University, Fukuoka 819-0395, Japan; 5 Faculty of Engineering, Department of Materials Science and Engineering, Kyushu University, Fukuoka 819-0395, Japan

## Abstract

It is extremely important to understand the properties of supported metal nanoparticles at the atomic scale. In particular, visualizing the interaction between nanoparticle and support, as well as the strain distribution within the particle is highly desirable. Lattice strain can affect catalytic activity, and therefore strain engineering via e.g. synthesis of core-shell nanoparticles or compositional segregation has been intensively studied. However, substrate-induced lattice strain has yet to be visualized directly. In this study, platinum nanoparticles decorated on graphitized carbon or tin oxide supports are investigated using spherical aberration-corrected scanning transmission electron microscopy (Cs-corrected STEM) coupled with geometric phase analysis (GPA). Local changes in lattice parameter are observed within the Pt nanoparticles and the strain distribution is mapped. This reveals that Pt nanoparticles on SnO_2_ are more highly strained than on carbon, especially in the region of atomic steps in the SnO_2_ lattice. These substrate-induced strain effects are also reproduced in density functional theory simulations, and related to catalytic oxygen reduction reaction activity. This study suggests that tailoring the catalytic activity of electrocatalyst nanoparticles via the strong metal-support interaction (SMSI) is possible. This technique also provides an experimental platform for improving our understanding of nanoparticles at the atomic scale.

Supported catalyst nanoparticles are critical components in energy technologies such as fuel cells, electrolyzers, and metal–air batteries. In polymer electrolyte membrane fuel cells (PEFCs), carbon black is generally used as an electrocatalyst support, due to the large surface area, and good electronic and thermal conductivity. Key goals are to lower catalyst loading, as well as improving the fundamental catalytic activity and durability^1^,^2^. Reducing the size of the Pt nanoparticles simultaneously increases electrochemical surface area (ECSA) and decreases loading. However, smaller nanoparticles generally have reduced lifetime in electrochemical devices; the weak interaction between platinum and carbon black leads to nanoparticle dissolution, aggregation and detachment during PEFC operation, leading to performance degradation. In addition, the high cathode potentials reached under PEFC start-stop operating conditions result in severe carbon corrosion and associated loss of ECSA over time. Therefore carbon-free support materials are desired to improve the durability of PEFC electrocatalysts^3^.

Several groups have proposed the use of metal oxides as alternative platinum catalyst supports with high resistance to electrochemical corrosion compared to carbon in PEFCs^4^,^5^. Our group has worked extensively on platinum-decorated tin oxide^6^. In particular Pt/SnO_2_ electrocatalysts can display remarkable durability under PEFC operating conditions compared with Pt/C^7^, indicating that carbon-free Pt/SnO_2_ electrocatalysts could potentially solve the problem of carbon corrosion. Platinum can bind to metal oxides much more strongly than to carbon, due to the strong metal-support interaction (SMSI)^8^. The result of this is that Pt nanoparticles are much less mobile on metal oxide supports compared with carbon, leading to improved durability^9^. Mukherjee *et al.* showed that in the case of 2 nm diameter Pt nanoparticles the influence of the support material on stability is significant^10^.

It has been shown that the electrochemical activity of nanoparticles can be enhanced on metal oxide supports^11^,^12^,^13^. For example, platinum monolayers on Au(111), Ir(111), Pd(111), Rh(111), and Ru(0001) surfaces have been shown to have different electrochemical oxygen reduction activities^14^. Mavrikakis *et al.* showed that there is a general correlation between surface strain and adsorption energies and activation energy barriers, attributed to a shift in the center of the metal d bands^15^. The above studies suggest that it may be possible to engineer the catalytic properties of nanoparticles via the SMSI effect. However, few studies have focused on the effect of substrate on the catalytic activity of platinum nanoparticles supported on carbon. Despite the importance of the catalyst-support interaction, and the effect that this may have on electrochemical durability and activity, detailed atomic-scale studies have not been widely performed on this system to date.

In practice such strain engineering is routinely achieved in Pt/C by alloying, or preparing core-shell structures^16^. The mechanism of activity enhancement in core–shell nanoparticles is attributed to the platinum-rich shell exhibiting compressive strain, resulting in a shift in the electronic band structure and an associated weakening of the strength of chemisorption in the oxidized species^1^. However, bimetallic core-shell systems degrade over time, resulting in poor stability^17^. Therefore, it is desirable to use strain to improve the fundamental catalytic activity, but without modifying the elemental composition of metal nanoparticle electrocatalyst. It is also widely reported that nanoparticle size strongly affects the fundamental activity of platinum electrocatalysts with a peak in mass activity at 2.2 nm. This has been related to strain^18^.

To fully understand the local behavior of supported nanoparticles on the atomic scale, more detailed analysis is crucial. Direct observational approaches are needed to investigate the interaction between nanoparticles and supports, and the lattice strain within the nanoparticles. The most common method for atomic-scale observation is transmission electron microscopy (TEM). However, observing local atomic strain or strained interactions between Pt particles and metal oxide supports is extremely challenging using conventional TEM with phase contrast imaging. Here, we use a Cs-corrected STEM coupled with GPA, supported by computational simulations in order to clarify the interaction between Pt nanoparticles and carbon or metal oxide supports. The aberration correction technique has made atom-by-atom observation of local atomic strain possible^19^. For example, Gan *et al.* have used this technique to demonstrate that a percolated lattice-contracted Pt−Fe core results in a gradient compressive strain in the Pt shell, providing direct evidence for the strain effect in enhanced ORR activity^20^. Prabhudev *et al.* have also performed atomic-scale two-dimensional surface relaxation mapping on similar PtFe core-shell electrocatalysts^21^.

Here, Pt nanoparticles with diameters of approximately 2 nm were decorated onto graphitized carbon (Pt/C) and tin oxide (Pt/SnO_2_) supports. Edge-on observation was chosen, and composition-resolved high angular annular dark field (HAADF) imaging was used. In HAADF the contrast is strongly dependent on atomic mass, resulting in clear cross sectional images of the interface in which the Pt and support can be clearly distinguished^22^.

Low resolution SEM and TEM images of Pt/C and Pt/SnO_2_ are shown in Fig. 1. TEM tomography was additionally performed to reveal the surface distribution of Pt nanoparticles on both supports (SI1 e, f). Pt is randomly dispersed across the support in both samples, with uniform particle size of 2 to 3 nm. Some minor agglomeration is observed on Pt/SnO_2_, which may indicate preferential deposition at atomic steps, as in the case of Au/TiO_2_^23^.

Cs-corrected STEM was used in order to observe the atomic structure in more detail, in particular at the interface between the nanoparticle and support. STEM has a particular advantage when imaging crystals, since dynamical diffraction-induced image contrast artifacts at interfaces are minimized. Therefore high resolution observation of individual atoms with enhanced Z contrast is possible. Figure 2a to c shows cross-sectional STEM-HAADF images of the Pt/C sample. The bright Pt nanoparticles stand out in high contrast to the dark grey carbon support and are generally spheroidal. Their unperturbed shape indicates that there is little interaction between the nanoparticle and the carbon support. The Pt lattice projection in Fig. 2b is determined from the atomic arrangement and spacing to be [110]. Lattice fringes can just be made out in the carbon support as alternating bands of light and dark contrast (indicated by dashed lines, Fig. 2b), suggesting that the surface is well-graphitized and displays negative curvature in the region of the nanoparticle.

Figure 2d and e show STEM-HAADF images of the Pt/SnO_2_ system. The lattices of the Pt nanoparticles and SnO_2_ support can both be clearly distinguished. In contrast to the carbon-supported Pt nanoparticles, the Pt nanoparticles decorated on SnO_2_ are consistently more *hemi*spherical in nature. Further examples of these hemispherical Pt nanoparticles are shown in the Supporting Information (Figure S1 and S3). Figure 2e,f show a Pt nanoparticle supported on SnO_2_, at higher magnification. This nanoparticle is located close to a facet edge. The projected Pt lattice is determined to be [031], whilst the SnO_2_ surface at the Pt/SnO_2_ interface is determined to be (100)^24^. An atomic step in the SnO_2_ terrace can be observed under the Pt nanoparticle, as indicated by the white arrow. The terrace edge corresponding to this step extends out of the plane of the image. Directly above this step, crystal lattice bending is clearly observed in the Pt nanoparticle. Such bending within the crystal lattice is intimately linked with the strain, and therefore could have implications for e.g. the catalytic activity, as discussed above. Therefore it is of interest to try and quantify and map the strain within the nanoparticle, and especially how it varies in the region of the atomic step.

In order to better understand distortions in the lattice, GPA was applied to the images in order to obtain lattice strain and lattice bending maps of the Pt nanoparticles. First, a fast Fourier transform (FFT) pattern was generated from the core region of the nanoparticle and the phase map was exported from a selected spot of the FFT. Next, a reference area was chosen in the region of the phase map with relatively little spatial change of phase. Finally, the map was calibrated based on the bulk lattice parameter of platinum. In this case, a dilatation map was used, which is the average of nominal strain components in the x and y directions. The values represented in the dilatation map represent the change in lattice parameter. The lattice parameter of bulk platinum is 2.265 nm (Powder Diffraction File PDF-2, International Center for Diffraction Data). According to direct observation in the STEM image (Fig. 2b), the lattice spacing at the center of the Pt nanoparticle is 0.24 nm. This suggests that the tensile strain at the center of the nanoparticle is 6%. The strain map is calibrated to this value, giving a reasonable estimation of the absolute tensile strain.

Figure 3a shows the resulting strain map overlaid on the original TEM image of the Pt/C system. The inset shows the absolute estimated lattice strain. In this nanoparticle, the lattice strain is remarkably homogeneous across the whole cross section of the nanoparticle. In other Pt nanoparticles, significant surface lattice expansion is observed, as shown in SI. This is in stark contrast to the strain map of the Pt/SnO_2_ system (Fig. 3b). Here, there is a strongly asymmetrical strain distribution across the surface and through the bulk of the Pt nanoparticle. The strain is greatest in the region above the atomic step (indicated by a white arrow) in the SnO_2_. The effects of this radiate out from the atomic step and permeate all the way to the surface of the Pt nanoparticle. However it must also be taken into account that this is a 2-dimensional projection of a 3-dimensional object, when interpreting these results. In future work we aim to map the strain in the platinum nanoparticles in three-dimensions, in order to further clarify the strain distribution.

These results are also supported by the crystal lattice bending map (Fig. 3c). The degree of lattice bending is plotted relative to a reference area at the center of the nanoparticle, where no lattice bending is observed. It has been frequently reported that at epitaxially grown layers that different lattice parameters cause bending towards the interface and in general the crystal lattice of Pt is expected to bend towards the source of strain^25^. Here, the Pt lattice bends towards the atomic step of the SnO_2_ substrate as highlighted in Fig. 3d. Therefore this atomic step is identified as the origin of lattice strain observed in the preceding strain maps. Recently it has been reported that Pt is strongly adsorbed at oxygen vacancy sites in TiO_2_^26^. Since TiO_2_ and SnO_2_ have similar crystal structure it is assumed that the strong lattice bending and the resulting strain are due to an oxygen vacancy site in the SnO_2_ lattice.

In particular, these experiments confirm that distortions in the crystal lattice of SnO_2_ induce strain in the supported Pt nanoparticle, and that this strain can propagate from the Pt/SnO_2_ interface all the way to the Pt surface. This was not observed in carbon-supported Pt nanoparticles. As previously discussed, lattice strain can strongly affect electrochemical activity^12^,^13^,^27^. Therefore it is expected that the support-induced lattice strain observed in this case could potentially improve catalytic activity.

In order to confirm these experimental observations and to better understand the interaction between the metal and support, we applied periodic plane wave density functional theory calculations to the two systems, implemented in the Vienna Ab Initio Software Package (VASP). The optimized geometry of a 74-atom Pt nanoparticle on a graphene surface is shown in Fig. 4a (side view) and 4b (top view). A single layer of graphene was used as a support in the simulation, for simplicity. All coordinates were fully relaxed, and bond lengths are given in Å. The graphene layer displays negative curvature, which is evidence for antibonding (i.e. a repulsive interaction between the nanoparticle and the support). Such antibonding-induced distortion of the carbon was not directly observed in the experimental images, since carbon black is comprised of multilayer carbon rather than single-layer graphene and is therefore much stiffer. However this repulsive force does explain the weak interaction between Pt and carbon, and has been explored in other studies^28^,^29^. As a result of this repulsive force, the geometry of the Pt nanoparticle is largely unperturbed by the presence of the support. The Pt-Pt bond lengths vary between 2.64 Å–2.79 Å (i.e. by 5.7%). Slightly shorter average bond lengths are observed at facet edges, whilst longer average bond lengths are observed on the facet planes. The optimized geometry of a Pt nanoparticle with 67 atoms was also calculated, adsorbed on the atomic step of a (100) SnO_2_ surface (Fig. 4c–d). The SnO_2_ surface was modelled with a two unit-cell layer thickness, where the coordinates of the first layer were kept fixed and those of the second layer were fully relaxed. The step has the (010) orientation. In this case the Pt-Pt bond length varies between 2.60 Å and 2.87 Å (i.e. 10.4%), over 80% more than in the Pt/C system. This is attributed to the effect of the atomic step in the support. This larger variation in bond lengths even propagates to the surface of the nanoparticle, in close agreement with the experimental results.

In order to explore a possible change in catalytic activity induced by strain in Pt nanoparticles, oxygen dissociation was investigated on unstrained, and strained Pt (111) surfaces, using DFT. Calculations were performed on a relaxed Pt surface, and a Pt surface strained by 3% in both lattice directions. The reaction mechanisms are depicted in Fig. 5a,b, and remain unchanged for both the relaxed and strained Pt surfaces. The oxygen molecule starts with top-bridge geometry and dissociates at a Pt atom. Finally the oxygen atoms reside in hollow positions of the Pt lattice. Although the mechanism is unchanged, the activation energy is significantly different: 0.52 eV for the relaxed surface, and 0.39 eV for the 3% strained surface. This result shows that 3% strain at the Pt(111) surface reduces the activation barrier for oxygen disassociation by 0.13 eV. The d-bands of the relaxed and strained Pt(111) surfaces are plotted in Fig. 5c,d, respectively. The d-bands are very similar, leading to the conclusion that the decrease in activation barrier is more likely a result of a steric effect on the surface rather than a change in electronic structure.

In conclusion, platinum-decorated carbon and tin oxide were synthesized and imaged at the atomic scale. The lattice strain was directly observed and mapped for the first time, using Cs-corrected STEM coupled with geometric phase analysis. The Pt formed spheroidal nanoparticles on carbon, whilst Pt hemispheres were formed on SnO_2_ due to the strong metal-support interaction. The variation in lattice parameter in the Pt/SnO_2_ system was significantly higher than that in Pt/C, especially near an atomic step in the SnO_2_ terrace. The resulting strain propagated to the surface of the nanoparticle. These systems were also modeled using DFT, and variations in strain throughout the Pt nanoparticles were observed in agreement with experiment. Further, oxygen dissociation was modelled on relaxed and strained Pt(111) surfaces. It was shown that the activation energy for oxygen disassociation was decreased when the Pt surface is under strain. These results suggest that it will be possible to tailor catalytic activity by taking advantage of the strong metal-support interaction between platinum and metal oxides. Strain engineering in this manner provides a simple alternative to the complex synthesis of e.g. bimetallic nanoparticles.

## Methods

Graphitized carbon black (Vulcan XC-72) and SnO_2_ (prepared by a co-precipitation method)^7^ were used as electrocatalyst supports. Platinum decoration was performed using platinum (II) acetylacetonate (Sigma-Aldrich Co. LLC, USA) as a precursor^30^. STEM images were acquired using a JEOL ARM-200F operating at 200 kV, equipped with a cold field emission gun (FEG) and a Cs-corrector (CEOS). The samples were mounted on carbon-coated copper grids (200 mesh, Ohken, Japan). A single fast scan was used to capture images in order to minimize any beam damage to the sample, which might affect the conclusions, however this results in some trade-off in image quality. In addition, imaging of the Pt/SnO_2_ samples was complicated by significant contamination due to outgassing. The electron beam acceleration was 200 kV, however the beam settings were restricted to a current of 25 pA by the use of a small convergent lens aperture and small probe size. Images were reconstructed from the TEM bright field image. A bespoke high-tilt sample holder was used (Mel-build, Japan), allowing the sample to be tilted by 45° to obtain a continuously tilted series of images for TEM tomography^31^. Strain field maps were obtained using dedicated software (Digital Micrograph, Gatan, US) for GPA of the STEM images. HAADF-STEM was used in order to obtain Z-contrast images^32^.

To investigate the strain across Pt/support heterojunctions, the in-plane strain distribution was calculated using GPA^33^ with respect to a region at the center of the same particle. In order to estimate the absolute lattice strain, this was calibrated to the bulk lattice parameter of platinum. The suitability of the reference area was checked by phase mapping, where the strain converged to a constant value. The results were used to construct false-color lattice strain maps associated with a specific lattice distortion component; specifically lattice deformation (dilatation/contraction), and lattice bending.

In the VASP, the Perdew, Burke, Ernzerhof (PBE-GGA) functional was employed using projector-augmented wave (PAW) pseudopotentials^34^,^35^,^36^,^37^,^38^,^39^. A cut-off energy of 300 eV and a k-point sampling of 2 × 2 × 1 were used to model the electronic properties of the surfaces. Pt surfaces made up of 4 layers were investigated. The bottom two layers were fixed and the top two layers were fully relaxed. The surfaces are terminated by 12 Å vacuum slabs. Calculations were performed with a 300 eV cut off energy and 3 × 3 × 1 k-points sampling using the Perdew–Burke–Ernzerhof (PBE) exchange-correlation functional. The reaction pathway was investigated using the nudged elastic band (NEB) method, implemented in VASP. Within the NEB method, five images were used to sample the band, including the starting and ending geometries. The transition states were confirmed using the climbing nudged elastic band (cNEB) method.

## Additional Information

**How to cite this article**: Daio, T. *et al.* Lattice Strain Mapping of Platinum Nanoparticles on Carbon and SnO_2_ Supports. *Sci. Rep.* **5**, 13126; doi: 10.1038/srep13126 (2015).

## Supplementary Material

Supplementary Figures

## Figures and Tables

**Figure 1:**
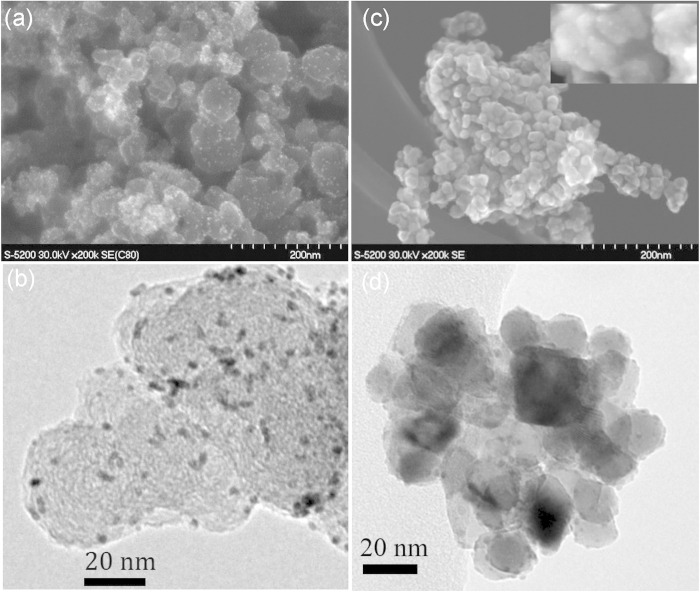
SEM images of (**a**) Pt/C and (**b**) Pt/SnO_2_. The inset image shows Pt nanoparticles on SnO_2_ at higher magnification. Typical TEM images of (**c**) Pt/C and (**d**) Pt/SnO_2_.

**Figure 2:**
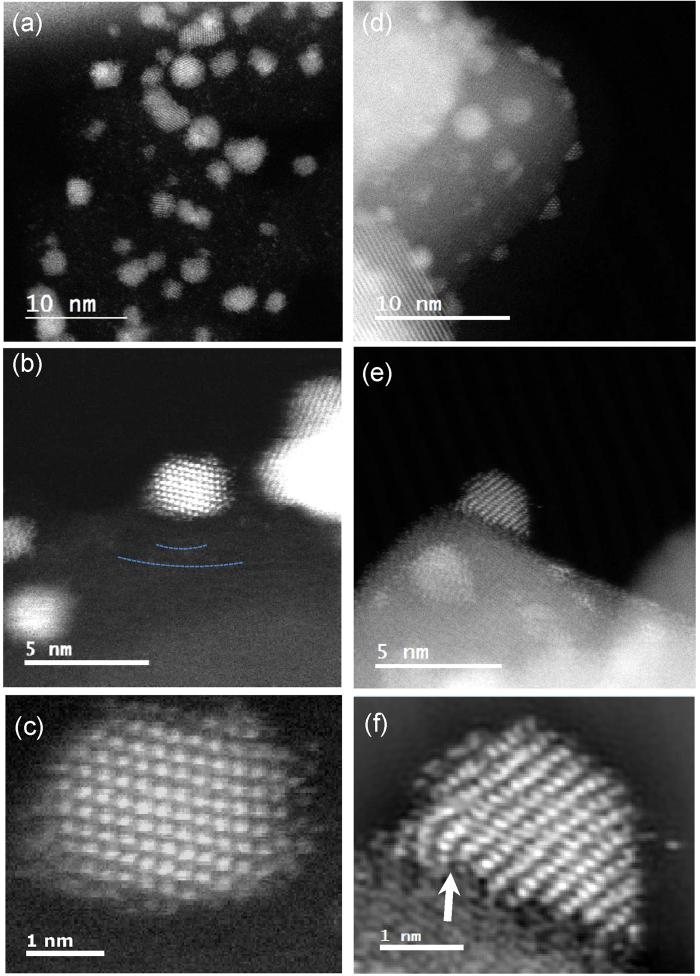
Atomic resolution STEM-HAADF cross-sectional images of (**a,b,c**) Pt/C and (**c,d,f**) Pt/SnO_2_. Brighter contrast corresponds to the higher Z-number of Pt atoms compared with SnO_2_ or carbon. In Fig. 2 (**c**), carbon lattice fringes with some degree of curvature are observed beneath the Pt nanoparticle, as marked by dashed lines.

**Figure 3:**
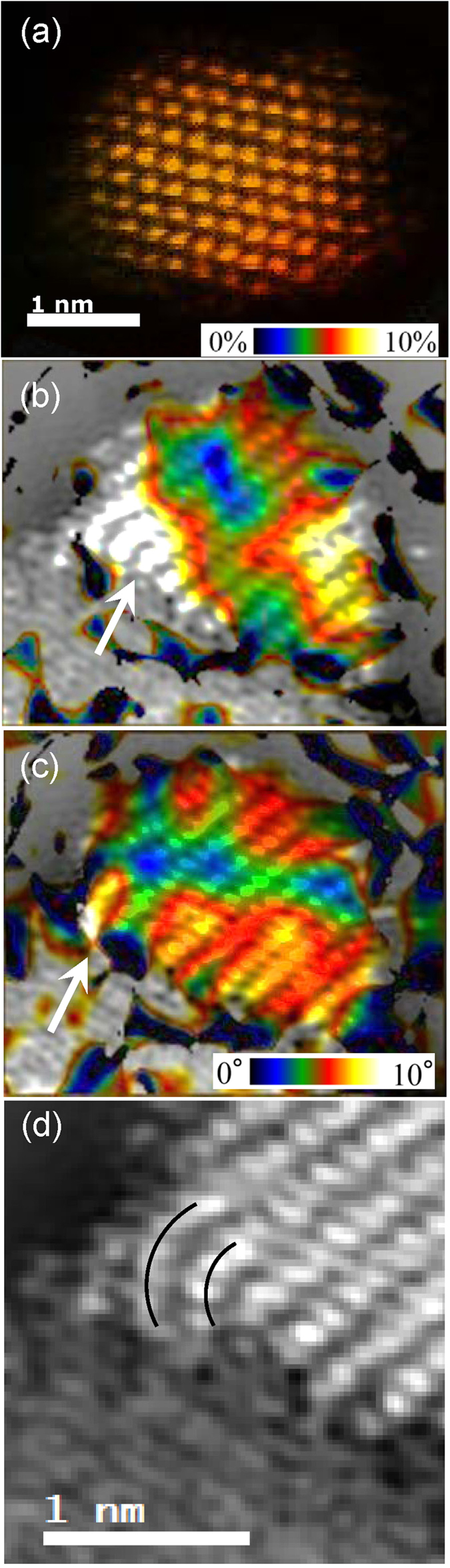
False-color lattice strain amplitude maps of (**a**) Pt/C, and (**b**) Pt/SnO_2_ obtained by GPA of the STEM-HAADF images. Strain maps were overlaid on the Cs-STEM image for clarity. (**c**) False-color lattice bending amplitude map for Pt/SnO_2_. (**d**) Strong lattice bending is observed in the region of the atomic step on the SnO_2_ surface.

**Figure 4:**
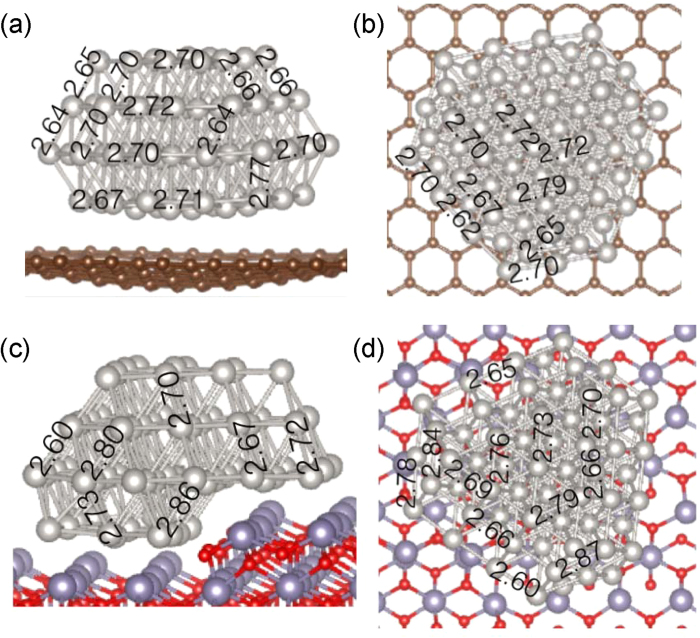
Computer simulated model by optimized geometry of a 74-atom Pt nanoparticle on a graphene surface. (**a**) Side view, and (**b**) top view. Simulation of the optimized geometry of a 67-atom Pt nanoparticle on a stepped SnO_2_ (100) surface, where the step has (0 1 0) orientation. (**c**) Side view, and (**d**) top view. All bond lengths are given in Å.

**Figure 5:**
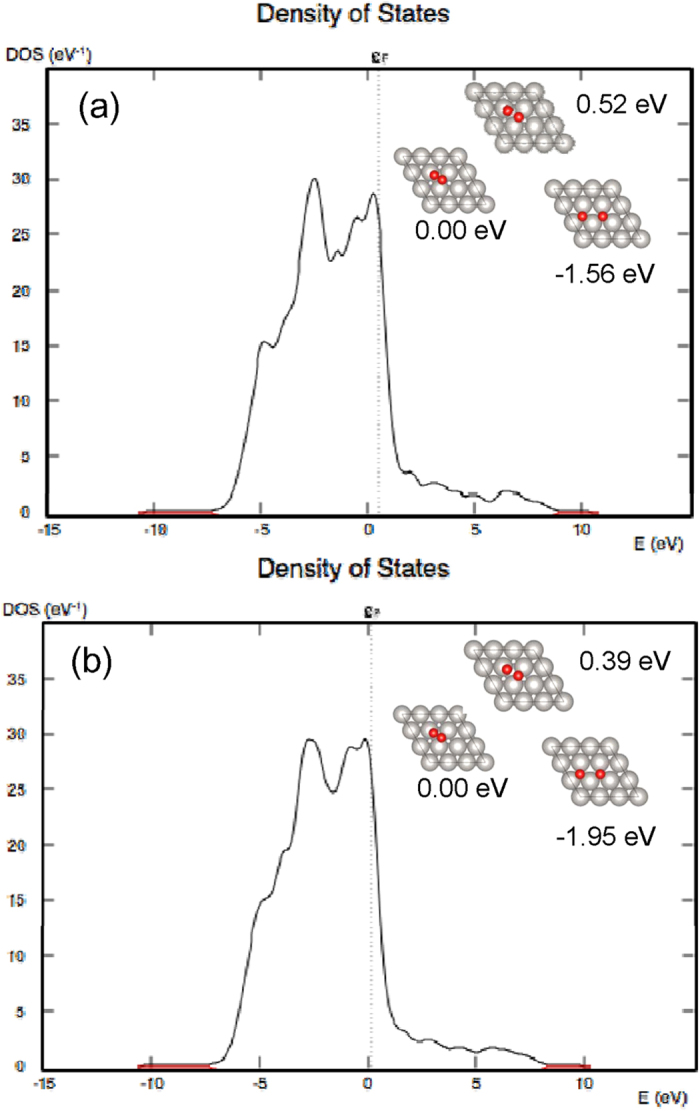
Computer simulated models of oxygen disassociation mechanisms on (**a**) relaxed and (**b**) strained Pt(111) surfaces. Simulated d-bands for (**c**) relaxed and (**d**) strained Pt(111) surfaces.
