# Supplementary material for: Lattice Strain Mapping of Platinum Nanoparticles on Carbon and SnO2 Supports
Source: Sci Rep. 2015 Aug 18;5:13126. doi: 10.1038/srep13126 (PMC4539540; doi:10.1038/srep13126)
Supplement: Supplementary Figures [file srep13126-s1.pdf]

Supplementary Information

Lattice Strain Mapping of Platinum Nanoparticles on Carbon and SnO_2 Supports

Takeshi DAIO[1,2,3*], Aleksandar STAYKOV[4], Limin GUO[4], Jianfeng LIU[4], Masaki Tanaka[5], Stephen Matthew LYTH[4], and Kazunari SASAKI[1,2,3,4*]

[1]International Research Center for Hydrogen Energy
[2] Next-Generation Fuel Cell Research Center (NEXT-FC)
[3] Faculty of Engineering, Department of Hydrogen Energy System
[4] International Institute for Carbon-Neutral Energy Research (WPI-I2CNER)
[5] Faculty of Engineering, Department of Materials Science and Engineering,
Kyushu University, Fukuoka 819-0395, Japan
*Correspondence to
[daio.takeshi.900@m.kyushu-u.ac.jp, sasaki.kazunari.278@m.kyushu-u.ac.jp]

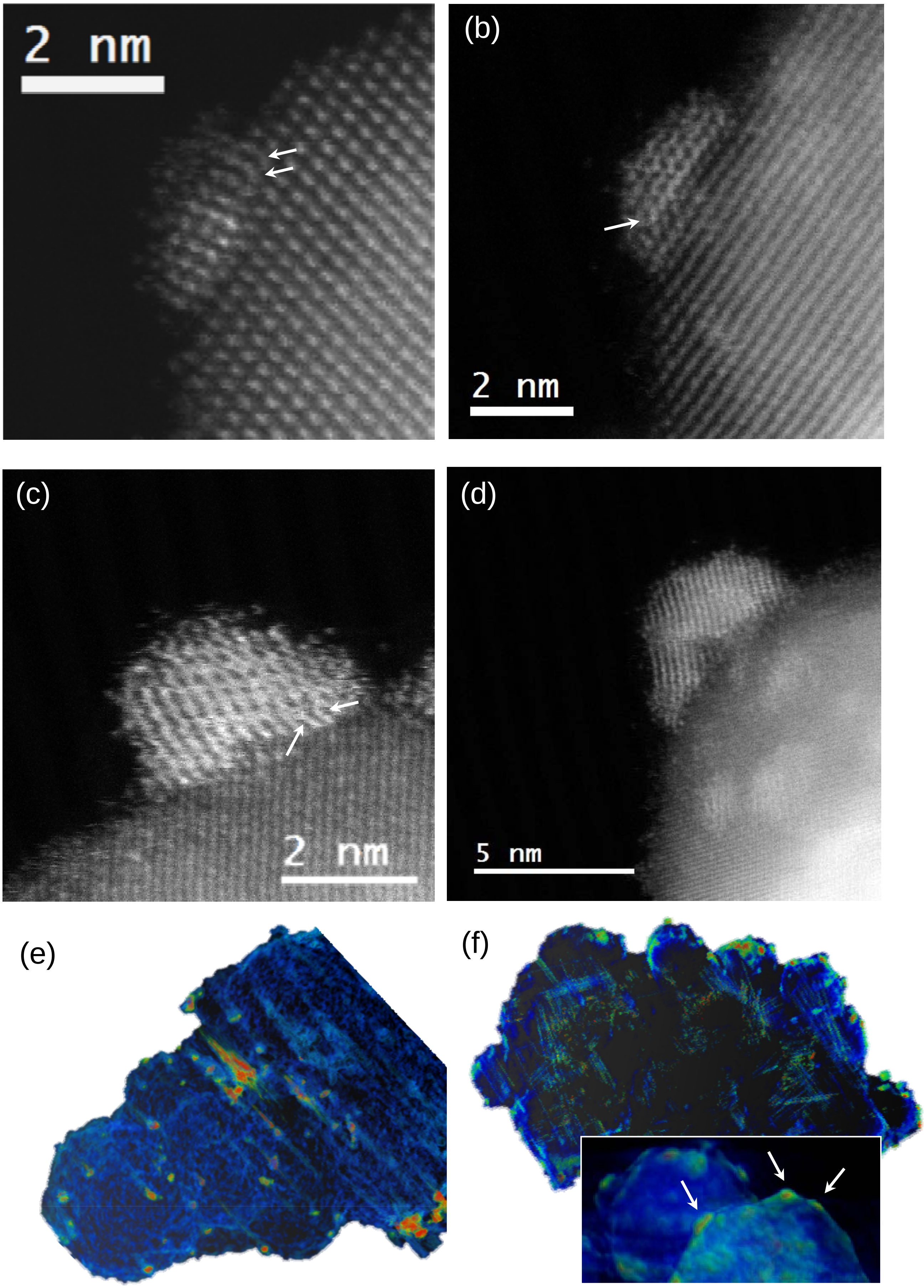
2 nm
(b)
2 nm
(c)
2 nm
(d)
5 nm
(e)
(f)

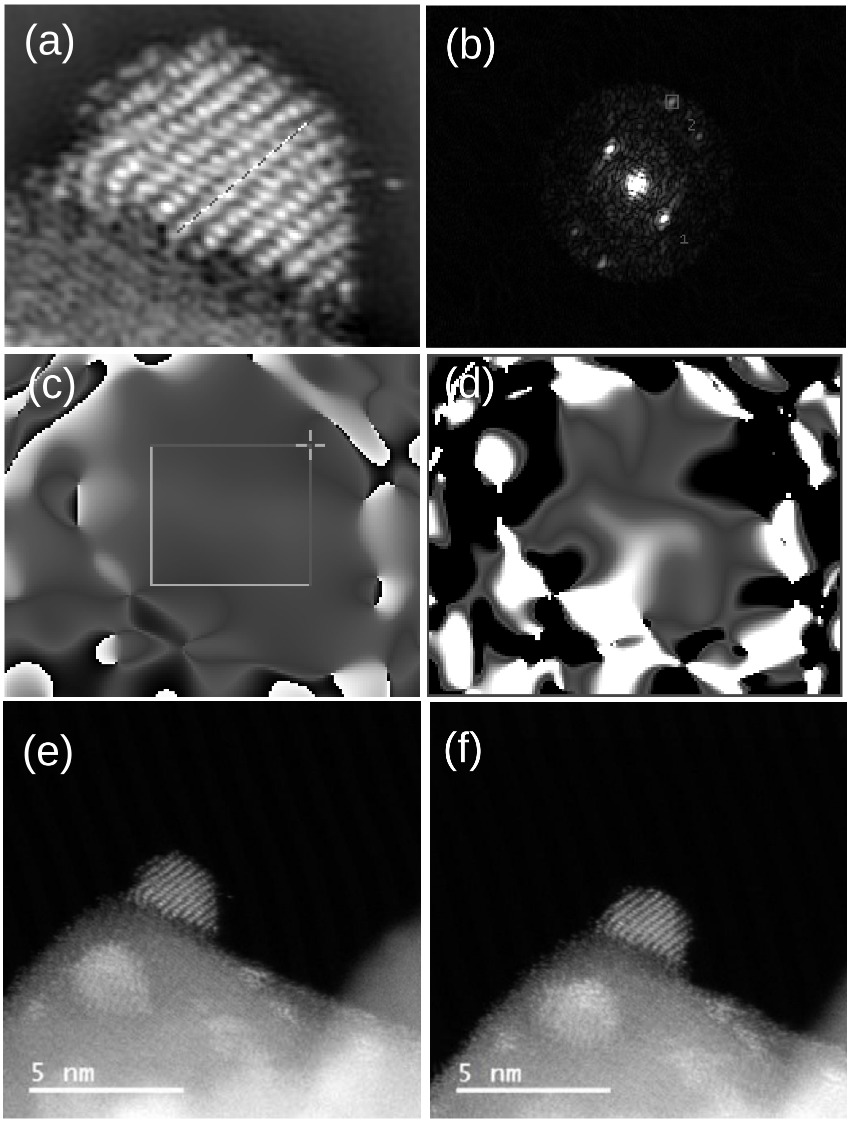
(a)
(b)
2
1
(c)
(d)
(e)
5 nm
(f)
5 nm

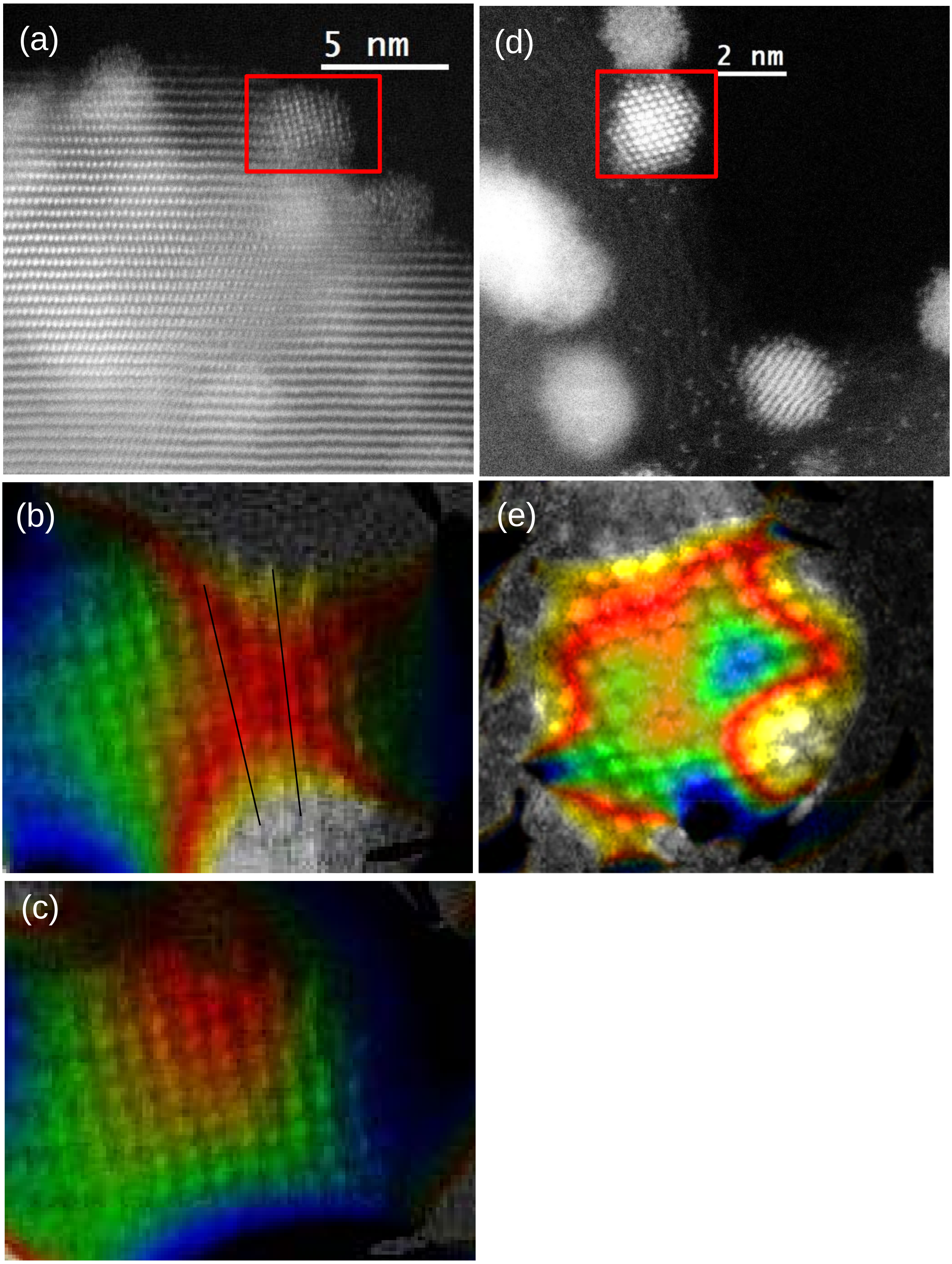
(a)
5 nm
(d)
2 nm
(b)
(e)
(c)
